# Work loss in patients with rheumatoid arthritis treated with abatacept, rituximab, tocilizumab or TNF inhibitors: a nationwide direct drug-to-drug comparison

**DOI:** 10.1136/rmdopen-2024-004936

**Published:** 2025-01-29

**Authors:** Gustaf Magnus Bruze, Thomas Frisell, Carl Turesson, Helena Forsblad-d'Elia, Jonas Soderling, Johan Askling, Martin Neovius, for the ARTIS Study Group, Gerd-Marie Alenius

**Affiliations:** 1 Clinical Epidemiology Division, Dept of Medicine, Karolinska Institutet, Stockholm, Sweden; 2 Rheumatology, Dept of Clinical Sciences, Malmö, Lund University, Malmö, Sweden; 3 Dept of Rheumatology and Inflammation Research, Sahlgrenska Academy, Univ. of Gothenburg, Gothenburg, Sweden; 4 Rheumatology Unit, Dept of Medicine, Karolinska Institutet, Stockholm, Sweden

**Keywords:** Rheumatoid Arthritis, Biological Therapy, Economics

## Abstract

**Objective:**

To compare work loss after starting tumour necrosis factor inhibitors (TNFi), rituximab, abatacept or tocilizumab in patients with rheumatoid arthritis (RA).

**Methods:**

We used data from the Swedish Rheumatology Quality Register to identify patients aged 19-62 years who were treated with TNFi (n=15 093), rituximab (n=2123), abatacept (n=1877) or tocilizumab (n=1720) between 2007 and 2020. Data on work loss (0–365 days per year) from sick leave and disability pension were retrieved from linkage to the Social Insurance Agency. Patients in the different treatment arms were balanced regarding baseline covariates using inverse probability weighting (IPTW).

**Results:**

Work loss increased for patients with RA until drug treatment initiation, reached a peak in the month after treatment initiation and then levelled off. Following IPTW, at 3 years before starting the treatment, there were no statistically significant differences in the mean annual adjusted work loss days between rituximab, abatacept or tocilizumab vs TNFi (mean difference vs TNFi: rituximab 1.1 days, 95% CI −4.5 to 6.7; abatacept 3.3, 95% CI −2.6 to 9.2; tocilizumab 1.2, 95% CI −4.9 to 7.3). At 3 years after starting the treatment (latest January 2021), there were also no statistically significant differences in the mean annual adjusted work loss days (mean difference: rituximab −4.8 days, 95% CI −11.3 to 1.7; abatacept 5.3, 95% CI −1.8 to 12.3; tocilizumab −0.6, 95% CI −7.7 to 6.5).

**Conclusions:**

Taking channelling into account, patients with RA treated with TNFi, rituximab, abatacept or tocilizumab had similar trajectories of work loss from sick leave and disability pension until treatment initiation, and similar trend breaks and plateau 3 years thereafter.

**WHAT IS ALREADY KNOWN ON THIS TOPIC**

- Rheumatoid arthritis (RA) is associated with significant costs to patients and society due to impaired work ability.

**WHAT THIS STUDY ADDS**

- This study compares the effectiveness of different bDMARDs (TNFi, rituximab, abatacept and tocilizumab) in terms of work loss (the sum of sick leave and disability pension).
- Our study describes the trajectories of work ability leading up to and following the start of treatment with bDMARDs.
- Taking channelling into account, patients with RA treated with different bDMARDs had similar trajectories of work loss before and after treatment initiation.

**HOW THIS STUDY MIGHT AFFECT RESEARCH, PRACTICE OR POLICY**

- While primary response, drug survival and other clinical metrics of treatment effectiveness are important, outcome metrics such as work ability are of critical importance for both patients and society.

## Introduction

 Besides an individual burden in terms of pain and morbidity, rheumatoid arthritis (RA) is associated with significant costs to the patient and society due to impaired work ability.^1^,^4^ Over the last decades, the introduction of new drugs has expanded the therapeutic armamentarium of disease-modifying anti-rheumatic drugs (DMARDs) against the disease and improved the chance of a favourable treatment outcome for the individual.

Recent EULAR and ACR treatment guidelines for RA list several bDMARDs, and more recently also JAK inhibitors, as viable drug treatment options and rank them as comparable once safety issues are accounted for.^5 6^ Although the average clinical effects (response rates) seem largely similar between biologic (bDMARDs) and targeted synthetic DMARDs (tsDMARDs),^7^,^11^ the extent to which the same applies also to other treatment outcomes, such as work loss, remains less well documented.

Previous studies on work ability in RA have compared the impact of strategies starting with TNFi vs triple therapy^12 13^ or tocilizumab vs methotrexate^14^ and described the longer-term work loss trajectories for TNFi initiators.^4^ Other RCTs and observational studies have examined the comparative effectiveness of different bDMARDs on clinical treatment outcomes (drug survival, good EULAR response, HAQ improvement, number of swollen or tender joints and CDAI remission).^7^,^11^ While these clinical treatment response measures are of interest to track disease progression, functional outcomes such as work ability are of great interest both to patients and to society. To date, however, there have been no studies with objectively measured data on work loss that have made a direct comparison of the effectiveness as defined by work ability/loss of all bDMARDs as used in RA.

The aim of this study was therefore to compare the effectiveness of bDMARDs as treatments for RA in a direct drug-to-drug comparison assessed by the impact of these drug treatments on work loss. We combine the number of days with compensation for sick leave and disability pension into one measure of work loss and follow patients with RA from 3 years before up to 3 years after starting the treatment, with data from nationwide Swedish registers and treatments administered and prescribed in a routine clinical setting.

## Methods

We identified patients with RA who started bDMARD treatment in the Swedish Rheumatology Quality (SRQ) Register. The register contains follow-up data for incident patients with RA and the Swedish Biologics Register ARTIS (1999–).^15^,^17^ Data from SRQ were linked with individual patient-level data from the Social Insurance Agency database MiDAS containing day-level information on sick leave and disability pension (2004–2021), using the personal identification number of each Swedish resident. Ethical approval was granted by the Regional Ethics Committee, Stockholm, Sweden.

### Setting

The Swedish healthcare system is tax-funded and offers universal access. Prescription drugs are provided free of charge above an annual threshold that is adjusted over time and was 2350 SEK (≈€230/$260) at the end of our study period. National treatment guidelines (largely adhering to the EULAR recommendations for the management of RA) guide the use of b/tsDMARDs in RA. The ultimate decision to treat with a b/tsDMARD resides with the treating rheumatologists; no formal applications or fulfilment of disease activity thresholds are required. In Sweden, patients with inflammatory rheumatic diseases are typically diagnosed and treated by rheumatologists rather than by general practitioners. Care for RA is provided in outpatient and inpatient facilities, and the vast majority of rheumatologists work at hospitals rather than as private practitioners.

The Swedish social insurance system provides compensation for sick leave and disability pension, both of which may be complete or partial. Data on work loss are recorded by the Swedish Social Insurance Agency. The retirement age in Sweden during our study period was generally 65 years, but with considerable individual variation. In this study, and to allow for up to 3 years of follow-up from treatment initiation until retirement age, the analyses were restricted to patients with RA aged 19–62 years at treatment initiation.

### Treatment initiator cohorts

**Figure 1:**
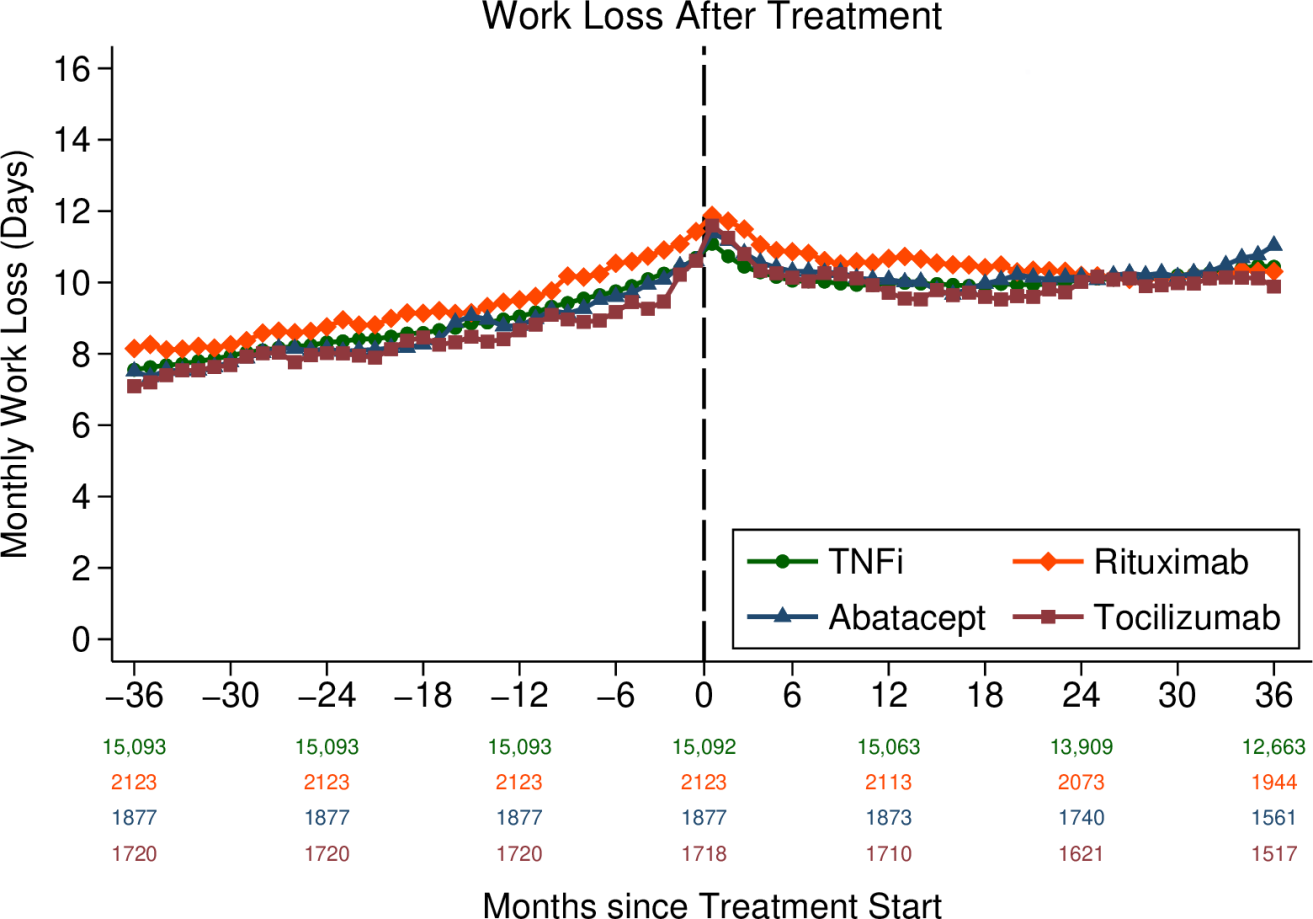
Mean inverse probability weighted monthly work loss days from 3 years before to 3 years after the treatment initiation for patients with rheumatoid arthritis receiving tumour necrosis factor inhibitors, rituximab, abatacept or tocilizumab. Numbers below x-axis are the number of observations for groups of drugs at different points in time. Observations are weighted by age, sex, education (three levels), year of treatment initiation, number of work loss days prior to treatment initiation, any work loss days (yes/no) prior to the treatment initiation and number of previous biologic drug treatments (zero, one, two, three or more).

As in our previous work,^7^ we identified four incident treatment exposure groups defined by initiating bDMARD treatments in the SRQ Register, with treatment initiation between 1 January 2007 and 31 January 2020: patients with RA who received, for the first time, (i) TNFi, (ii) rituximab, (iii) abatacept or (iv) tocilizumab. The four groups contain treatment episodes, and a patient may be included in more than one treatment group (if the patient received more than one specific bDMARD drug treatment during the study period). Patients were followed until the end of follow-up (31 January 2021), death or emigration, whichever occurred first. Our end cut-off date for inclusion of patients ensures that we can have at least 1 year of data on sick leave and disability pension after treatment initiation for all treatment episodes in our sample. We initially included also tsDMARDs (JAKis), but as they were introduced later in the study period (median treatment initiation year 2019 vs 2012–2014 for the other drugs) and therefore had a considerably shorter follow-up, they were excluded from further analyses (online supplemental table 1 and figure 1).

To study transitions in and out of work loss, we identified two separate groups of treatment episodes. The first cohort (the incidence of work loss cohort) consists of treatment episodes for patients with no work loss (0 days) 3 months before treatment initiation. A second cohort (the remission of work loss cohort) consists of treatment episodes for patients with full sick leave (90 days) and no disability pension (0 days) 3 months before treatment initiation. The rationale for not including patients with a disability pension in the remission of the work loss cohort was that disability pension reflects more permanent disability, any changes to which may also be subject to greater administrative inertia than sick leave.

### Outcome

The primary outcome was net annual compensated days of work loss, defined as the extent of sick leave or disability pension times the number of days with the benefit. For example, one net day of work loss may be equal to 1 day of 100% sick leave, 2 days with 50% sick leave or 1 day of 50% sick leave and 1 day of 50% disability pension. Net annual compensated days of work loss could range between 0 days (the minimum) and 365 days (the maximum).

In the analysis of transitions in and out of work loss, an event for patients in the incidence of work loss cohort was a month with 15 or more days of work loss. In the remission of work loss cohort, an event was defined as a month with 15 or fewer days of work loss.

#### Sick leave

During the study period, the first day of a sick leave episode was unpaid, and compensation for the second to 14th day of a sick leave episode was paid by the employer. As a consequence, only episodes of sick leave exceeding 14 days were recorded in the Social Insurance Agency database and included in this study.

#### Disability pension

Disability pension consists of ‘sickness compensation’ (‘sjukersättning’) for individuals aged 30–64 years and ‘activity compensation’ (‘aktivitetsersättning’) for individuals aged 19–29 years. Both sickness and activity compensation may be time-limited or permanent and require a 25% or more reduction in work ability, expected to last for at least 1 year.

### Data sources

Data on drug use were obtained from SRQ with an estimated coverage of bDMARD treatment of 90%^17^ and the national Prescribed Drug Register with close to complete national coverage.

Data on medical history before treatment initiation was retrieved from the National Patient Register with inpatient and non-primary-care outpatient visits (serious infection, diabetes, chronic obstructive lung disease, heart failure, stroke and number of days hospitalised). Data on recent and non-recent malignancies were obtained from the National Cancer Register. Medical history variables describe events during the 5 year period before treatment initiation, except for serious infection which describes diagnoses during 1 year period before treatment initiation, and non-recent malignancy which describes malignancies that occurred more than 5 years before treatment initiation.

Data on the educational level was obtained from the Swedish Longitudinal Integration Database for Health Insurance and Labour Market Studies (LISA),^18^ which covers the adult population in Sweden, and educational level was classified into three categories (primary school, high school, university).

### Subgroup analysis

In subgroup analysis, we investigated the net annual compensated days of work loss in groups defined by patients’ educational attainment (three categories: retrieved from the LISA register at Statistics Sweden), patients’ previous number of biological drug treatments (four categories: from SRQ and the Prescribed Drug Register) and patients’ visual analogue scale (VAS) pain at the onset of treatment (four equally large groups of treatments defined from quartiles of self-reported pain in SRQ). We also conducted a subgroup analysis restricted to the group of patients who remained on the same drug throughout the whole 3 year follow-up period.

### Statistics

Statistical analyses were performed using SAS (version 9.4, SAS Institute Inc., Cary, NC, USA) and Stata (version 13.1, College Station, TX). The main summary measure was the mean annual days of work loss. We also calculated median days and the percent of participants with different levels of work loss each year. Unless otherwise stated, all the analyses were conducted with an intention-to-treat protocol, that is, were not censored at any treatment discontinuation (which would lead to informative censoring).

For the main analysis, the mean days per year were calculated counting from 3 years before the day of treatment initiation until up to 3 years after the day of treatment initiation. We also conducted a more detailed analysis by month from 36 months before to 36 months after the day of treatment initiation.

The mean adjusted difference in annual days of work loss between the four groups of drug treatments was estimated with linear regression. Time-to-event analyses of time to first work loss episode (the incidence of work loss cohort) and first work episode (the remission of work loss cohort), respectively, were conducted using Cox proportional hazard models.

To achieve balance in pre-treatment covariates between patients in the four groups of drugs, work loss data from individual treatment episodes were weighted with stabilised inverse probability weighting (IPTW). These weights were estimated with propensity scores from a multinomial logistic regression model and defined as the inverse of the predicted probability of receiving each drug treatment multiplied by the fraction of patients receiving the treatment. The covariates used in the multinomial model were age, sex, education (three levels), the year of treatment initiation, the number of work loss days during 1, 2 and 3 years before the treatment initiation, a dichotomous variable (0/1) indicating if an individual received any work loss compensation in 1, 2 or 3 years before the treatment initiation, and the number of previous biologic drug treatments (zero, one, two, three or more).

For the subgroup analyses, we estimated new stabilised inverse probability weights using estimated propensity scores from a multinomial logistic regression model (the same method as in the main analysis). In the subgroup analysis for education, we excluded educational attainment from the set of covariates used to estimate inverse probability weights. In the subgroup analysis for the number of previous biological drug treatments, we excluded the number of previous biological drug treatments from the covariates.

Linear regression models and Cox proportional hazard models were weighted by the stabilised inverse probability weights and further adjusted for age, sex, education, duration of RA and the number of previous biologic drug treatments (double robust estimation). For both models, we computed robust standard errors clustered at the level of the individual patient.

A p value of <0.05 was considered statistically significant.

### Patient and public involvement

Patients and/or the public were not involved in the design, conduct, reporting, or dissemination plans of this research.

## Results

We identified a total of 20 813 drug treatment initiations in SRQ, (TNFi n=15 093, rituximab n=2123, abatacept n=1877 and tocilizumab n=1720) (table 1). The inverse probability weighted mean age of treatment initiation was around 48 years for the different treatment groups, the year of treatment initiation spanned from 2007 to 2020 with the median treatment initiation in 2013 or 2014 and a majority of treated patients were women. Patients had received an average of slightly more than one previous bDMARD drug treatments before the relevant bDMARD treatment initiation, had been diagnosed with RA 9 to 11 years prior to treatment initiation depending on the treatment group and had similar educational levels across treatment groups. Unweighted characteristics are presented in online supplemental table 1.

**Table 1:** Inverse probability weighted characteristics of patients with rheumatoid arthritis at the treatment initiation

	TNFi	Rituximab	Abatacept	Tocilizumab
	(n=15 093)	(n=2123)	(n=1877)	(n=1720)
Start year				
Median	2013	2013	2014	2014
(p25-p75)	(2010–2017)	(2011–2016)	(2012–2016)	(2012–2016)
Demographics				
Age (years)	48.3 (10.4)	48.8 (9.9)	47.9 (10.6)	48.0 (10.4)
Women (%)	80.1	78.2	76.6	80.3
RA clinical characteristics				
Previous biologic drug treatments	1.1 (1.5)	1.1 (1.5)	1.1 (1.4)	1.1 (1.4)
0 (%)	46.3	45.1	44.8	45.3
1 (%)	25.2	24.4	24.1	24.5
2 (%)	13.9	13.5	13.9	13.8
≥3 (%)	14.8	15.0	15.0	14.7
RA duration (years)	10.3 (9.2)	11.3 (8.8)	8.8 (8.1)	8.7 (8.5)
HAQ	1.0 (0.6)	1.1 (0.6)	1.1 (0.6)	1.2 (0.6)
DAS28	4.6 (1.4)	4.8 (1.3)	4.8 (1.3)	5.0 (1.3)
VAS pain	56.0 (24.5)	56.6 (23.6)	57.4 (23.7)	60.0 (23.9)
Work loss (days)*				
Work loss (days)	10.7 (13.1)	11.4 (13.2)	10.6 (13.0)	10.6 (13.0)
Sick leave (days)	4.7 (9.8)	4.9 (9.8)	4.8 (9.6)	4.9 (9.9)
Disability pension (days)	5.9 (10.9)	6.5 (11.2)	5.8 (10.8)	5.7 (10.8)
Education				
Primary school (%)	14.5	15.2	14.5	14.6
High school (%)	48.7	48.5	48.5	47.0
University (%)	36.6	36.0	36.7	38.1
Education missing (%)	0.3	0.4	0.3	0.2
Medical history†				
Serious infection (%)	2.6	4.9	4.7	2.2
Recent malignancy (%)	2.0	6.2	2.1	1.4
Nonrecent malignancy (%)	6.1	9.2	5.1	5.0
COPD (%)	1.0	2.1	2.6	2.3
Diabetes (%)	4.9	6.1	5.4	4.5
Stroke (%)	0.5	0.8	0.7	0.5
Heart failure, (%)	0.5	1.3	2.6	1.0
Days hospitalised	5.7 (20.6)	8.2 (21.2)	6.5 (20.8)	6.1 (20.6)

Values are mean (SD) unless otherwise stated. obstructive pulmonary disease. Each observation is a treatment episode. All characteristics at baseline are weighted by standardizedstandardised inverse probability weights.

* During the month before treatment startinitiation.

† Medical history variables describe events during the five5 year period before treatment startinitiation, except for serious infection, which describes diagnoses during the one1 year period before the treatment startinitiation, and nonrecent malignancy which that describes malignancies that occurred more than five5 years before the treatment startinitiation.

COPDChronic obstructive pulmonary diseaseDAS28Disease Activity Score 28HAQHealth Assessment QuestionnaireRArheumatoid arthritisVASvisual analogue scale

### Work loss

The weighted mean days of work loss per month increased in all four treatment groups during 3 years leading up to the treatment initiation. Mean days of work loss days reached their peak during the month after the treatment initiation and then remained (as opposed to continued to increase) at a fairly constant level during 3 years of follow-up (figure 1). A separate analysis of sick leave and disability pension (online supplemental figure 2 and 3, respectively) showed that days of sick leave induced most of this variation over time while days with disability pension increased steadily in all four treatment groups. Unweighted mean days of work loss are presented in online supplemental figure 4.

Following IPTW weighting, during the 1 year period starting 3 years before the treatment initiation, there was no statistically significant difference in the mean adjusted annual days of work loss between rituximab, abatacept or tocilizumab vs TNFi (rituximab 1.1 days, 95% CI −4.5 to 6.7; abatacept 3.3, 95% CI −2.6 to 9.2; tocilizumab 1.2, 95% CI −4.9 to 7.3). Similarly, there was no statistically significant difference in the mean adjusted days of work loss between the treatment groups in the third year after treatment initiation (rituximab −4.8 days, 95% CI −11.3 to 1.7; abatacept 5.3, 95% CI −1.8 to 12.3; tocilizumab −0.6, 95% CI −7.7 to 6.5; (reference TNFi); online supplemental table 2).

**Figure 2:**
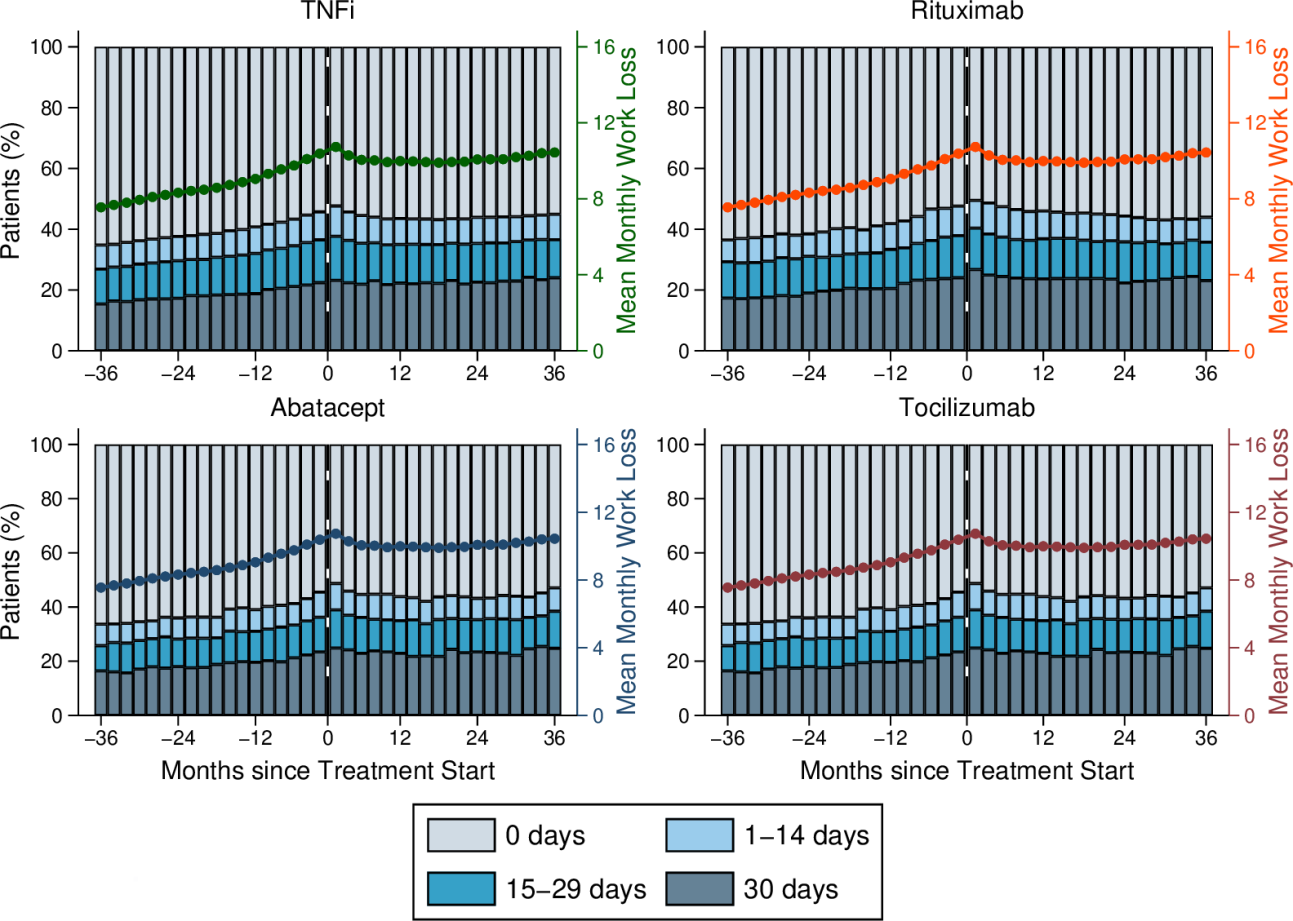
Distribution of mean monthly work loss days. Mean monthly work loss days for treatment episodes with tumour necrosis factor inhibitors (top left), rituximab (top right), abatacept (bottom left) and tocilizumab (bottom right). Solid line is weighted mean monthly work loss days. Bars show weighted percentage of treatment episodes by category of mean monthly work loss days (maximum 30 days).

We also analysed the weighted distribution of monthly work loss days before and after the treatment in the four treatment groups by categorising monthly work loss episodes into 0 days of work loss, 1–14 days of work loss, 15 to 29 days of work loss and 30 days of work loss (the maximum corresponding to full work loss). The proportion with no work loss fell in all four treatment groups during the time period leading up to the treatment initiation and then remained fairly constant (figure 2). By analogy, the proportion with full work loss (30 days per month) increased before the treatment initiation and then remained fairly constant.

### Subgroup results

#### Educational level

In subgroup analysis, we considered the evolution of the mean work loss days per month in patient groups defined by educational attainment. The mean work loss days were lower in patients with university education, but there were no significant differences between treatment groups among patients with university education. Among patients without a university degree, those treated with abatacept had significantly higher work loss days 3 years after treatment (online supplemental figure 5 and table 3).

#### Previous bDMARD drug treatments

The number of previous biological drug treatments had a large and significant impact on the mean work loss days, with higher treatment order being associated with more mean work loss days (online supplemental figure 1 and table 4).

#### VAS pain

Self-reported VAS pain at the onset of drug treatment had a large and significant impact on the mean work loss days, with higher self-reported pain being associated with more days of work loss (online supplemental figure 6 and table 5).

#### Patients remaining on drug

Our main analysis was conducted with an intention-to-treat protocol where we followed patients from 3 years before to up to 3 years after the day of treatment initiation, irrespective of changes in drug treatment during this period. At 3 years after treatment initiation, between 32% and 48% of patients remained on the same drug (table 2). As a subgroup analysis, we considered the subset of patients who remained on the same drug for the whole 3 year follow-up period. The trajectories for work loss before and after treatment initiation in this analysis were similar to the trajectories in the intention-to-treat analysis (online supplemental figure 7).

**Table 2:** Unweighted number of patients remaining on the same drug after the treatment initiation

	TNFi	Rituximab	Abatacept	Tocilizumab
	(n=15 093)	(n=2123)	(n=1877)	(n=1720)
On treatment				
1 year, n (%)	9711 (64.3)	1589 (74.8)	1152 (61.4)	1045 (60.8)
2 year, n (%)	7204 (47.7)	1267 (59.7)	797 (42.5)	780 (45.3)
3 year, n (%)	5572 (36.9)	1022 (48.1)	607 (32.3)	631 (36.7)

Unweighted number of patients remaining on the same drug, one 1, two2, or three3 years after the treatment initiation. Data from the Swedish Rheumatology Quality Register, including the Swedish Biologics Register ARTIS.

### Incidence of work loss

**Figure 3:**
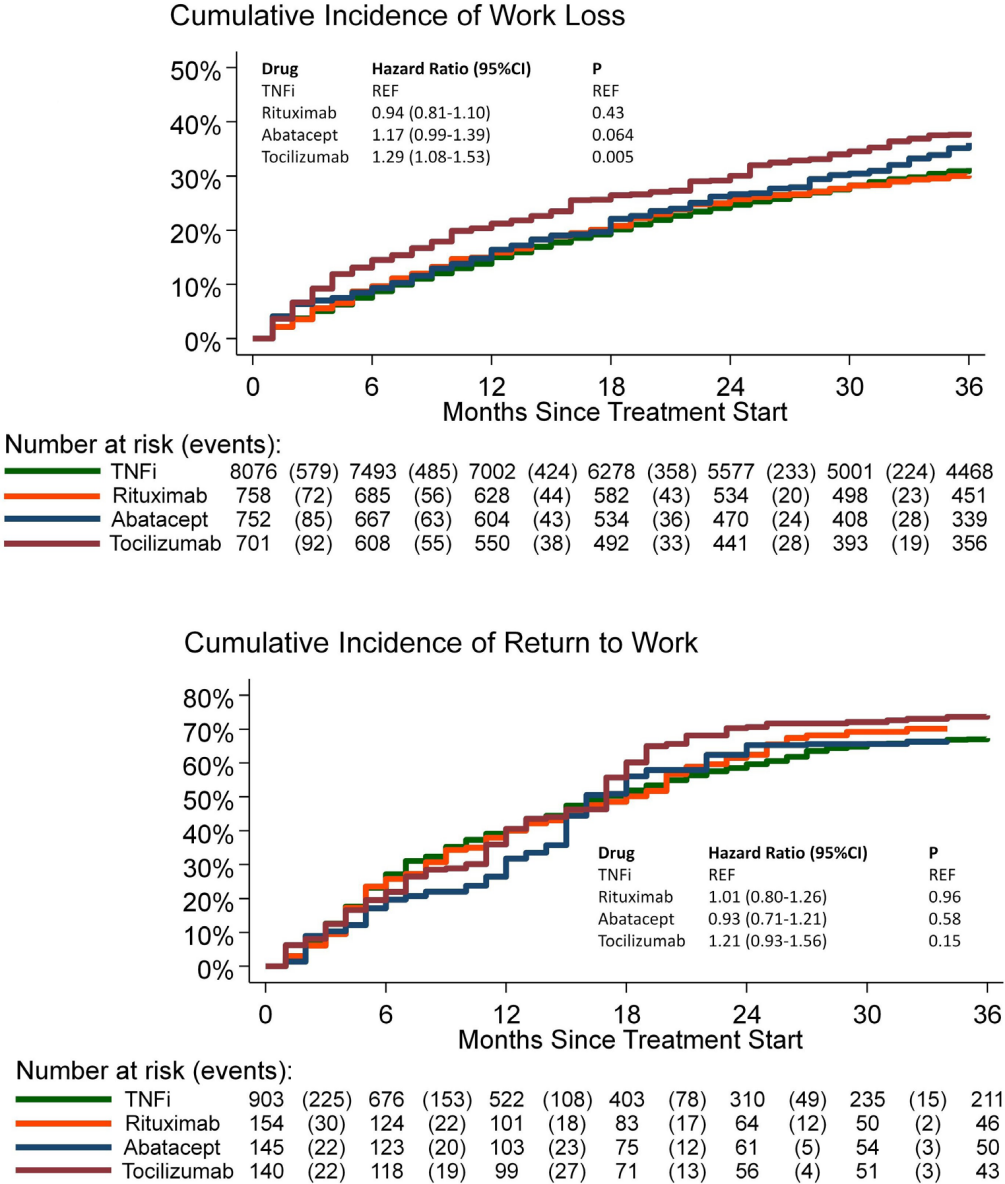
Incidence of work loss (top) and incidence of remission of work loss (bottom). In the incidence cohort (top), an event is a month with 15 or more work loss days. In the remission cohort (bottom), an event is a month with 15 or less work loss days. Cumulative incidence functions are inverse probability weighted by age, sex, education (three levels), year of treatment initiation, number of work loss days prior to treatment initiation, any work loss days (yes/no) prior to treatment initiation and number of previous biologic drug treatments (zero, one, two, three or more).

We identified a total of 10 287 treatments (TNFi n=8076, rituximab n=758, abatacept n=752 and tocilizumab n=701) in the incidence of work loss cohort for patients with no (0 days) work loss during the 3 months before the treatment initiation. The inverse probability weighted cumulative incidence of work loss was 15% (TNFi), 16% (rituximab and abatacept) and 21% (tocilizumab) at 1 year and 31% (TNFi), 30% (rituximab), 36% (abatacept) and 38% (tocilizumab) at 3 years after the treatment initiation . The adjusted HR showed a higher incidence of work loss among patients receiving tocilizumab relative to TNFi (adjusted HR 1.29, 95% CI 1.08 to 1.53 figure 3) but no statistically significant difference for rituximab vs TNFi (adjusted HR 0.94, 95% CI 0.81 to 1.10), or for abatacept vs TNFi (adjusted HR 1.17, 95% CI 0.99 to 1.39).

### Remission of work loss

We identified a total of 1342 treatments (TNFi n=903, rituximab n=154, abatacept n=145 and tocilizumab n=140) in the remission of work loss cohort for patients with full sick leave (90 days) and no disability pension (0 days) during the 3 months before the treatment initiation. The inverse probability weighted cumulative incidence for the remission of work loss at 1 year after the treatment initiation was 41% (TNFi), 40% (rituximab), 41% (tocilizumab) and 32% (abatacept), and at 3 years after treatment initiation 67% (TNFi), 70% (rituximab), 67% (abatacept) and 74% (tocilizumab). Treatment episodes with tocilizumab had the numerically highest adjusted HR, but none of the adjusted HRs for the remission of work loss were statistically significant (tocilizumab vs TNFi adjusted HR 1.21, 95% CI 0.93 to 1.56; rituximab vs TNFi 1.01, 95% CI 0.80 to 1.26; abatacept vs TNFi 0.93, 95% CI 0.71 to 1.21; figure 3).

## Discussion

We performed a direct drug-to-drug comparison in routine clinical practice regarding work loss after the initiation of four different bDMARDs, using Swedish day-level registry data on sick leave and disability pension. Work loss increased for patients with RA up until the treatment initiation and then levelled off. There was no statistically significant difference in the mean adjusted work loss days per year between patients in the treatment groups, neither at 3 years before nor at 3 years after the treatment initiation. This result was present also in patients remaining on the same drug for 3 years of follow-up, suggesting that the lack of association between a particular drug treatment and mean work loss days is not induced by patients who switched drugs during follow-up.

Some previous studies have compared the impact on work loss of different bDMARD or tsDMARD treatments vs conventional sDMARD treatment in patients with RA. These studies include the Swedish randomised drug trial SWEFOT comparing the effectiveness of infliximab vs conventional combination treatment for patients with early RA (reporting no statistically significant differences over 2^12^ or 7^13^ years), and the Dutch randomised drug trial U-Act-Early comparing tocilizumab, methotrexate or their combination for patients with early RA (reporting no statistically significant differences over 2 years).^14^ Our current study is the first larger study that uses objectively measured data on work loss to make a direct comparison of the effectiveness of bDMARD treatments on work loss for patients with RA.

In the current study, mean monthly work loss increased for each successive month during the 36 months prior to bDMARD start, and thereafter, it decreased marginally and levelled off. This is similar to previously reported Swedish work loss data in patients with RA initiating TNFi:s 1999–2007.^4^ At the time of bDMARD initiation, about 20% of patients in the current study were on full-time sick-leave or disability pension. This can be compared with 30–35% of patients in the study of TNFi initiators from 1999 to 2007, despite similar disease duration and HAQ scores.^4^ In both the current and the previous study, the percentage on full-time sick-leave or disability pension remained largely constant over the 3 and 5 years of follow-up in the respective studies.

Our study is one of the first comparative effectiveness studies of different bDMARDs with respect to work loss among RA patients. The main strength of our study is that it is based on nationwide registry data from routine clinical practice, which ensures broad coverage and a high generalisability of the results as well as high accuracy in our data regarding the administration of different groups of drugs and the reporting of sick-leave and disability pension.

The main weaknesses are that our data on sick leave do not include sick-leave episodes shorter than 14 days and that our study is an observational study as opposed to a randomised controlled trial, which means that treatment groups may not be fully balanced. We used IPTW and achieved similar levels and trends in work loss before the treatment initiation across our four treatment groups, as well as balance in disease-specific, demographic, socio-economic and other measured variables. Randomised trials of sufficient size are likely to also balance unmeasured variables, while we do not know how well our design managed to account for unmeasured characteristics. We also did not include JAKis in this analysis; a direct comparison vs these drugs will require that enough patients have accrued reasonably long follow-up time for the outcomes under study.

In conclusion, among patients with RA treated with various bDMARDs, work loss days from sick-leave and disability pension followed similar trajectories of increasing work loss during the 3 years leading up to the treatment initiation, and for all treatment arms, a similar trend break and levelling off in work loss was observed for 3 years thereafter.

## supplementary material

10.1136/rmdopen-2024-004936 online supplemental file 1

## Data Availability

No data are available.
