# Supplementary material for: Work loss in patients with rheumatoid arthritis treated with abatacept, rituximab, tocilizumab or TNF inhibitors: a nationwide direct drug-to-drug comparison
Source: RMD Open. 2025 Jan 29;11(1):e004936. doi: 10.1136/rmdopen-2024-004936 (PMC11867670; doi:10.1136/rmdopen-2024-004936)
Supplement: online supplemental file 1 [file rmdopen-11-1-s001.pdf]

Supplementary Appendix

Work loss in patients with rheumatoid arthritis treated with abatacept, rituximab, tocilizumab or TNF inhibitors: a nationwide cohort study

G Bruze[1], T Frisell[1], C Turesson[2], H Forsblad-d'Elia[3], J Söderling[1], J Askling[1,4], M Neovius[1]
for the ARTIS Study Group

[1]Clinical Epidemiology Division, Dept of Medicine, Karolinska Institutet, Stockholm, Sweden
[2]Rheumatology, Dept of Clinical Sciences, Malmö, Lund University, Malmö, Sweden
[3]Dept of Rheumatology and Inflammation Research, Sahlgrenska Academy, University of Gothenburg, Gothenburg, Sweden
[4]Rheumatology Unit, Dept of Medicine, Karolinska Institutet, Stockholm, Sweden

Table of Contents

Item	Description	Page
Table S1	Unweighted characteristics of patients with RA at treatment initiation	2
Table S2	Mean adjusted work loss days	3
Table S3	Mean adjusted work loss days and educational attainment	4
Table S4	Mean adjusted work loss days and treatment order	5
Table S5	Mean adjusted work loss days and self-reported pain	6
Figure S1	Mean work loss days and treatment order	7
Figure S2	Mean sick leave days	8
Figure S3	Mean days with receipt of disability pension	9
Figure S4	Unweighted mean work loss days	10
Figure S5	Mean work loss days and educational attainment	11
Figure S6	Mean work loss days and self-reported pain	12
Figure S7	Mean work loss days for patients on the same drug	13

Supplementary Tables

Table S1 Unweighted characteristics of patients with RA at treatment initiation

	JAKi (n=859)	TNFi (n=15,093)	Rituximab (n=2123)	Abatacept (n=1877)	Tocilizumab (n=1720)
Start-year					
Median (p25-p75)	2019 (2018-2020)	2013 (2010-2016)	2012 (2010-2015)	2014 (2012-2017)	2014 (2012-2016)
Demographics					
Age (years)	48.8 (10.0)	47.7 (10.6)	50.8 (9.0)	49.7 (9.7)	48.7 (10.1)
Women, n (%)	83.5	11,913 (78.9)	1709 (80.5)	1560 (83.1)	1417 (82.4)
RA clinical characteristics					
Previous biologic drug treatments	2.2 (1.7)	0.8 (1.2)	1.8 (1.6)	2.1 (1.6)	2.2 (1.6)
0 (%)	132 (15.4)	8690 (57.6)	483 (22.8)	248 (13.2)	225 (13.1)
1 (%)	199 (23.2)	3799 (25.2)	566 (26.7)	460 (24.5)	423 (24.6)
2 (%)	199 (23.2)	1446 (9.6)	496 (23.4)	507 (27.0)	451 (26.2)
≥3 (%)	329 (38.3)	1158 (7.7)	578 (27.2)	662 (35.3)	621 (36.1)
RA duration (years)	9.8 (7.6)	9.5 (9.2)	12.6 (9.0)	11.6 (8.7)	11.3 (8.8)
HAQ	1.1 (0.7)	1.0 (0.6)	1.2 (0.6)	1.2 (0.6)	1.3 (0.6)
DAS28	4.5 (1.4)	4.5 (1.4)	5.0 (1.3)	4.8 (1.4)	5.1 (1.4)
VAS pain	58.4 (24.8)	55.0 (24.6)	59.1 (23.4)	60.5 (23.1)	62.1 (23.5)
Work loss (days)*					
Work loss (days)	8.4 (12.3)	9.5 (12.7)	14.8 (13.7)	13.2 (13.6)	12.8 (13.5)
Sick leave (days)	4.9 (3.5)	4.5 (9.6)	5.1 (10.0)	5.4 (10.2)	5.7 (10.5)
Disability pension (days)	3.5 (8.9)	5.0 (10.2)	9.7 (12.8)	7.8 (12.1)	7.1 (11.8)
Education					
Primary school, n (%)	103 (12.0)	2115 (14.0)	362 (17.1)	274 (14.6)	235 (13.7)
High school, n (%)	435 (50.6)	7203 (47.7)	1070 (50.4)	954 (50.8)	887 (51.6)
University, n (%)	317 (36.9)	5738 (38.0)	683 (32.2)	642 (34.2)	593 (34.5)
Education missing, n (%)	*4 (0.5)*	*37 (0.2)*	*8 (0.4)*	*7 (0.4)*	*5 (0.3)*
Medical history**					
Serious infection, n (%)	18 (2.1)	342 (2.3)	118 (5.6)	104 (5.5)	54 (3.1)
Recent malignancy, n (%)	18 (2.1)	286 (1.9)	126 (5.9)	51 (2.7)	33 (1.9)
Nonrecent malignancy, n (%)	61 (7.1)	870 (5.8)	179 (8.4)	112 (6.0)	101 (5.9)
COPD, n (%)	16 (1.9)	131 (0.9)	49 (2.3)	40 (2.1)	29 (1.7)
Diabetes, n (%)	50 (5.8)	713 (4.7)	155 (7.3)	130 (6.9)	98 (5.7)
Stroke, n (%)	3 (0.4)	62 (0.4)	19 (0.9)	13 (0.7)	11 (0.6)
Heart failure, n (%)	8 (0.9)	66 (0.4)	31 (1.5)	37 (2.0)	23 (1.3)
Days hospitalized	3.7 (13.3)	4.9 (19.3)	10.6 (26.0)	7.4 (19.3)	7.0 (20.1)

Values are mean (SD) unless otherwise stated. COPD=Chronic obstructive pulmonary disease. Each observation is a treatment episode.

*During month before treatment start.

**Medical history variables describe events during the five-year period before treatment start, except for serious infection which describes diagnoses during the one-year period before treatment start, and nonrecent malignancy which describes malignancies that occurred more than five years before treatment start.

Table S2 Adjusted mean difference in work loss days per year before and after treatment initiation

	**Difference vs TNFi**	
	**Estimate**	**95% CI**
Work Loss 3yrs Before		
Rituximab	1.09	(-4.48, 6.65)
Abatacept	3.29	(-2.60, 9.17)
Tocilizumab	1.17	(-4.93, 7.27)
TNFi	Reference	
Work Loss 3yrs After		
Rituximab	-4.80	(-11.29, 1.68)
Abatacept	5.28	(-1.76, 12.31)
Tocilizumab	-0.58	(-7.69, 6.53)
TNFi	Reference	

Mean difference in work loss days per year relative to patients receiving TNF inhibitors, at three years before and three years after treatment initiation (adjusted for age, sex, education in three categories, duration of RA, and number of previous drug treatments, and weighted by the inverse probability of receiving the drug treatment). Reference group is patients receiving TNF inhibitors.

Table S3 Adjusted mean difference in work loss days per year before and after treatment initiation and educational level

	Difference vs TNFi	
	Estimate	95% CI
WITH UNIVERSITY EDUCATION		
Work Loss 3yrs Before		
Rituximab	-1.17	(-8.67, 6.34)
Abatacept	-2.03	(-9.75, 5.68)
Tocilizumab	3.88	(-4.12, 11.88)
TNFi	Reference	
Work Loss 3yrs After		
Rituximab	2.14	(-7.36, 11.64)
Abatacept	-6.36	(-16.41, 3.68)
Tocilizumab	4.47	(-5.60, 14.54)
TNFi	Reference	
WITHOUT UNIVERSITY EDUCATION		
Work Loss 3yrs Before		
Rituximab	2.49	(-5.01, 10.00)
Abatacept	7.54	(-0.53,15.61)
Tocilizumab	-0.26	(-8.64, 8.13)
TNFi	Reference	
Work Loss 3yrs After		
Rituximab	-8.02	(-16.56, 0.52)
Abatacept	13.87	(4.47, 23.26)
Tocilizumab	-3.58	(-13.14, 5.97)
TNFi	Reference	

Mean difference in work loss days per year relative to patients receiving TNF inhibitors, at three years before and three years after treatment initiation in subgroups of patients with or without university education (adjusted for age, sex, duration of RA, and number of previous drug treatments, and weighted by the inverse probability of receiving the drug treatment). Reference group is patients receiving TNF inhibitors.

Table S4 Adjusted mean difference in work loss days per year before and after treatment initiation and treatment order

	Difference vs 1st drug treatment	
	Estimate	95% CI
Work Loss 3yrs Before		
1st drug treatment	Reference	
2nd drug treatment	22.67	(18.55,26.79)
3rd drug treatment	43.77	(38.64,48.89)
4th or more drug treatment	67.38	(62.26,72.51)
Work Loss 3yrs After		
1st drug treatment	Reference	
2nd drug treatment	26.02	(21.04,31.00)
3rd drug treatment	44.60	(38.46,50.73)
4th or more drug treatment	74.80	(68.67,80.92)

Mean difference in work loss days per year relative to patients with no previous drug treatments, at three years before and three years after treatment initiation (adjusted for age, sex, duration of RA and education, and weighted by the inverse probability of receiving the drug treatment). Reference group is patients with no previous drug treatments.

Table S5 Adjusted mean difference in work loss days per year before and after treatment initiation and self-reported pain at treatment initiation

	Difference vs 1st quartile	
	Estimate	95% CI
Work Loss 3yrs Before		
1st quartile	Reference	
2nd quartile	20.44	(14.69, 26.18)
3rd quartile	29.22	(23.48, 34.97)
4th quartile	42.26	(36.51, 48.00)
Work Loss 3yrs After		
1st quartile	Reference	
2nd quartile	29.15	(22.41, 35.90)
3rd quartile	47.07	(40.32, 53.82)
4th quartile	67.58	(60.85, 74.31)

Mean difference in work loss days per year relative to patients with self-reported pain in lowest quartile, at three years before and three years after treatment initiation (adjusted for age, sex, duration of RA and education, and weighted by the inverse probability of receiving the drug treatment). Reference group is patients with self-reported pain in the lowest quartile.

Supplementary Figures

BY NUMBER OF PREVIOUS DRUGS

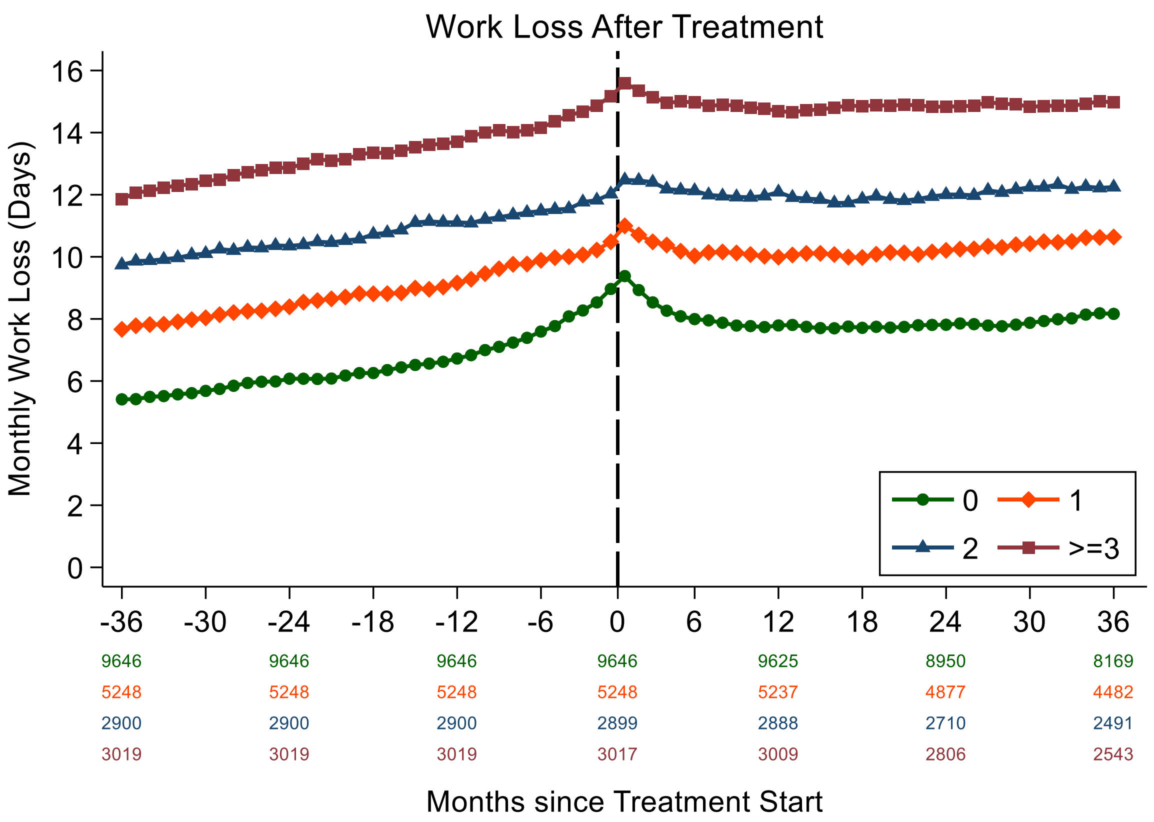

Figure S1 Mean inverse probability weighted monthly work loss in days for patients with RA from three years before to three years after treatment initialisation and number of previous biologic drug treatments (zero, one, two, three or more).

Numbers below x-axis are number of observations for groups of drugs at different points in time.

Observations are weighted by age, sex, education (three levels) and year of treatment initiation.

SICK LEAVE ONLY

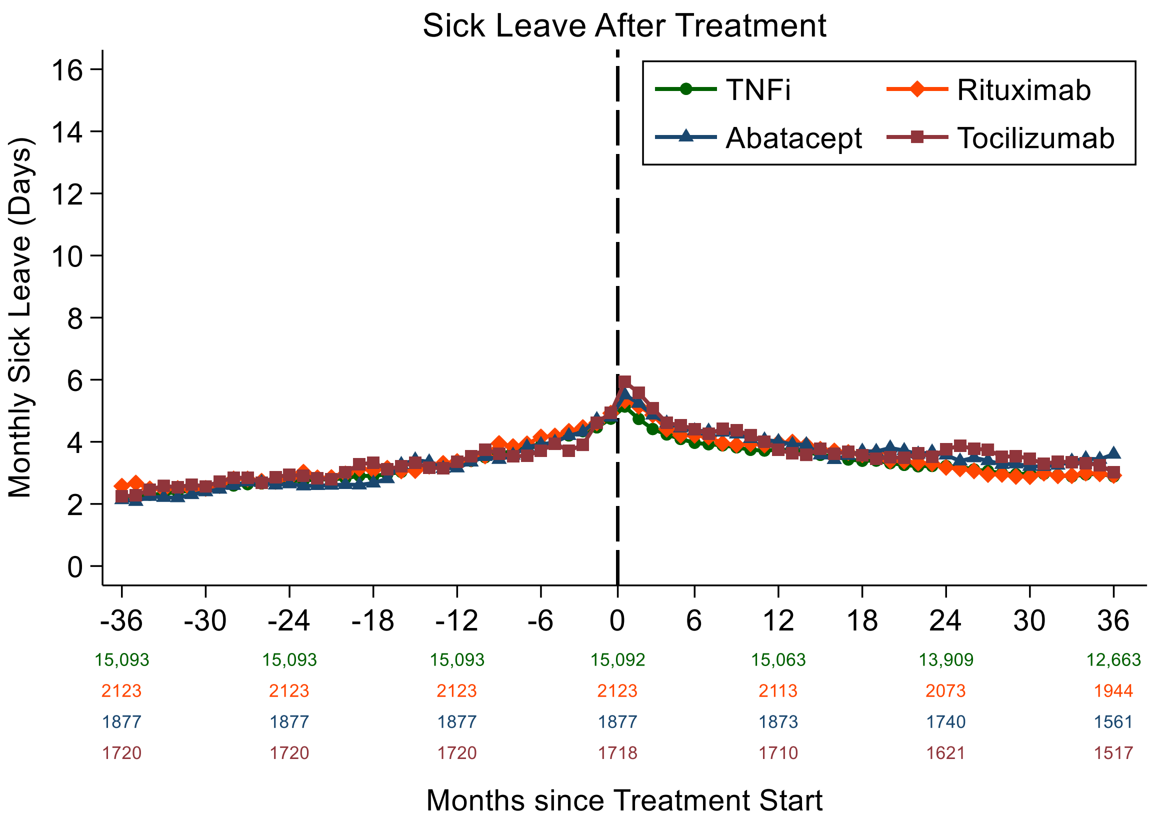

Figure S2 Mean inverse probability weighted monthly sick leave days for patients with RA from three years before to three years after treatment initialisation.

Numbers below x-axis are number of observations for groups of drugs at different points in time.

Observations are weighted by age, sex, education (three levels), year of treatment initiation, number of work loss days during one, two, or three years prior to treatment initiation, whether or not patient had any work loss days (yes/no) during one, two, or three years prior to treatment initiation, and number of previous biologic drug treatments (zero, one, two, three or more).

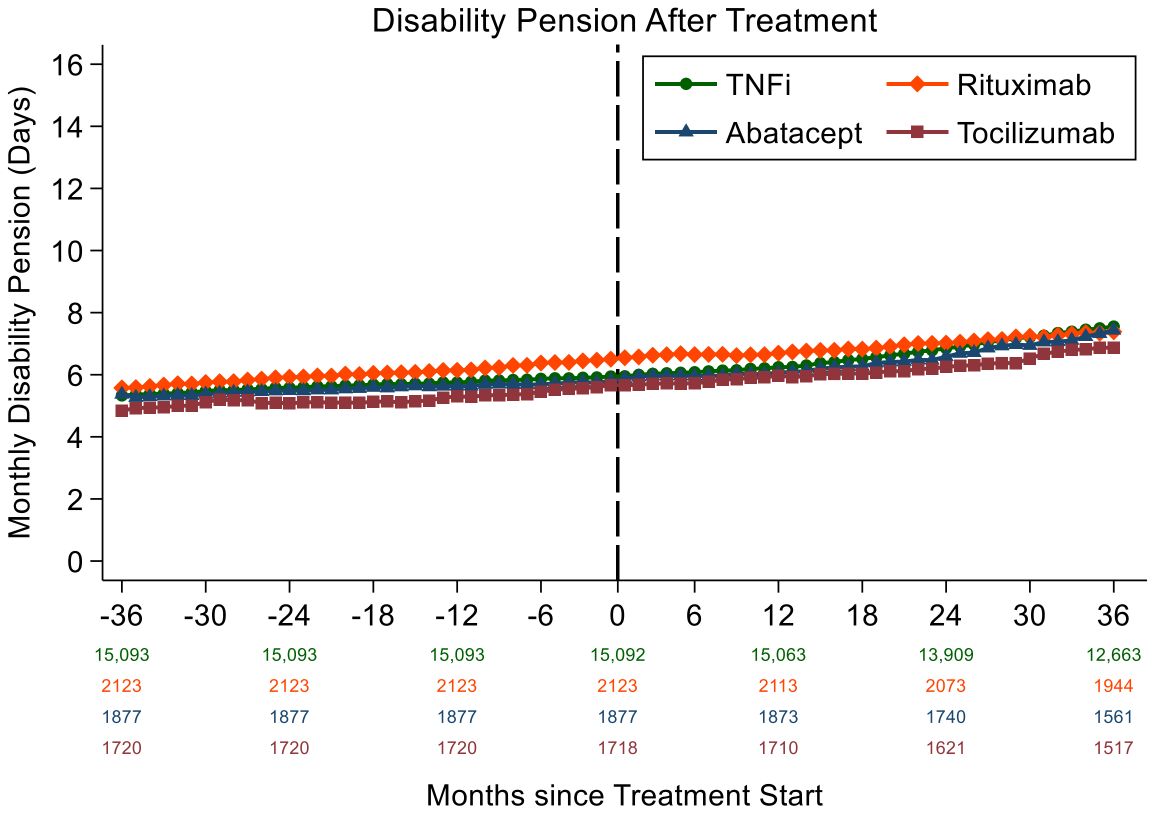

Figure S3 Mean inverse probability weighted monthly days with disability pension for patients with RA from three years before to three years after treatment initialisation.

Numbers below x-axis are number of observations for groups of drugs at different points in time.

Observations are weighted by age, sex, education (three levels), year of treatment initiation, number of work loss days during one, two, or three years prior to treatment initiation, whether or not patient had any work loss days (yes/no) during one, two, or three years prior to treatment initiation, and number of previous biologic drug treatments (zero, one, two, three or more).

UNWEIGHTED

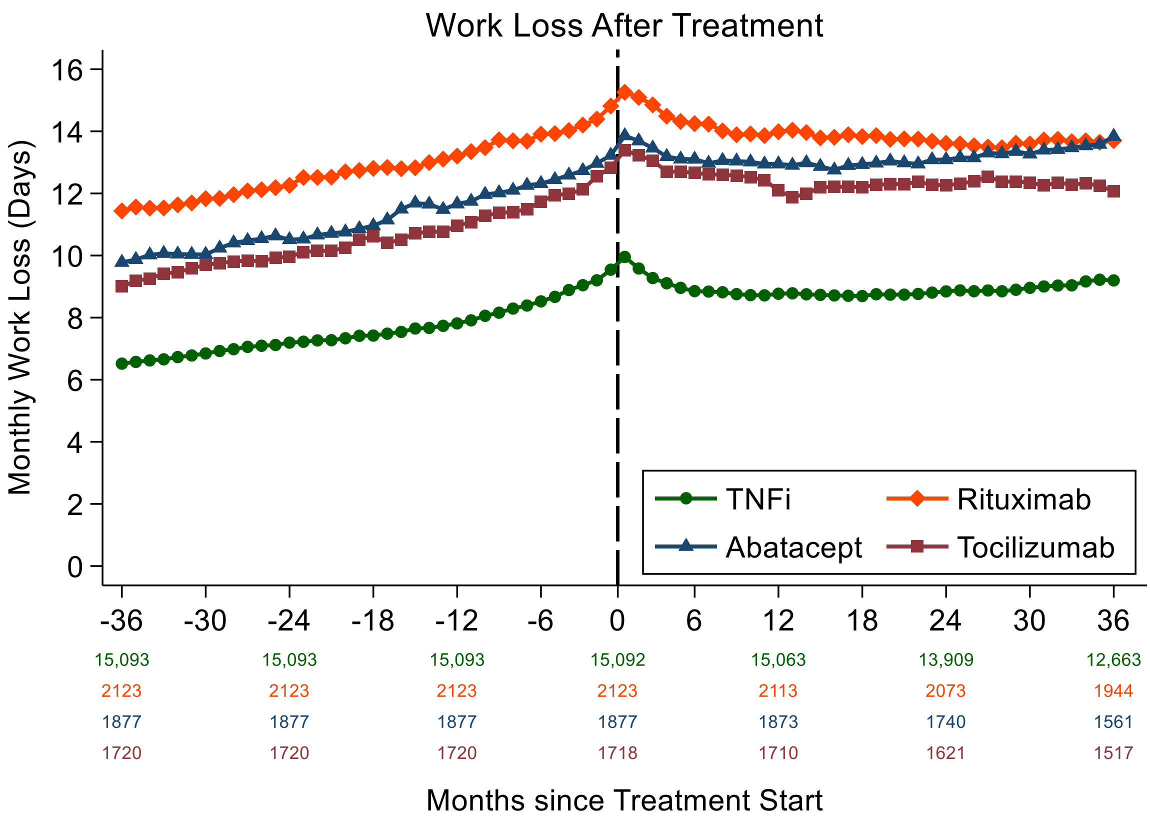

Figure S4 Crude monthly work loss in days for patients with RA from three years before to three years after treatment initialisation and previous biologic drug treatments (zero, one, two, three or more).

Numbers below x-axis are number of observations for groups of drugs at different points in time.

WITH UNIVERSITY EDUCATION

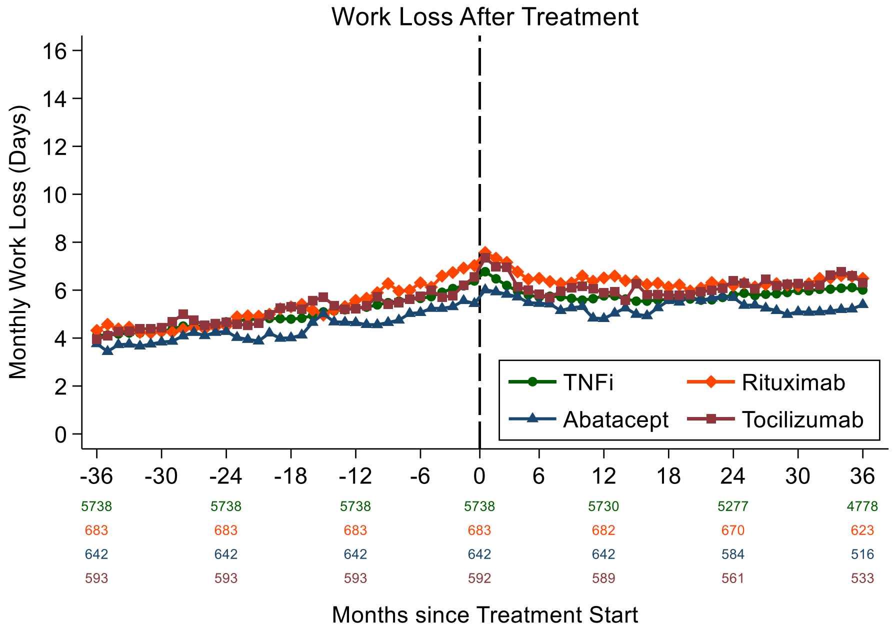

WITHOUT UNIVERSITY EDUCATION

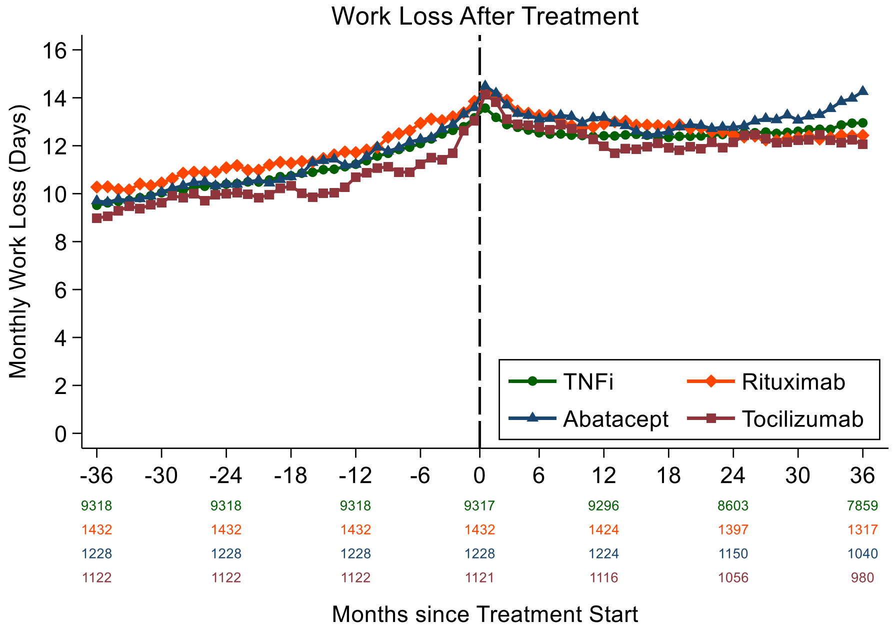

Figure S5 Mean inverse probability weighted monthly work loss in days for patients with RA from 3 years before to 3 years after treatment initialisation with (top) and without (bottom) a university degree.

Numbers below x-axis are number of observations for groups of drugs at different points in time.

Observations are weighted by age, sex, year of treatment initiation, number of work loss days prior to treatment initiation, any work loss days (yes/no) prior to treatment initiation, and number of previous biologic drug treatments (zero, one, two, three or more).

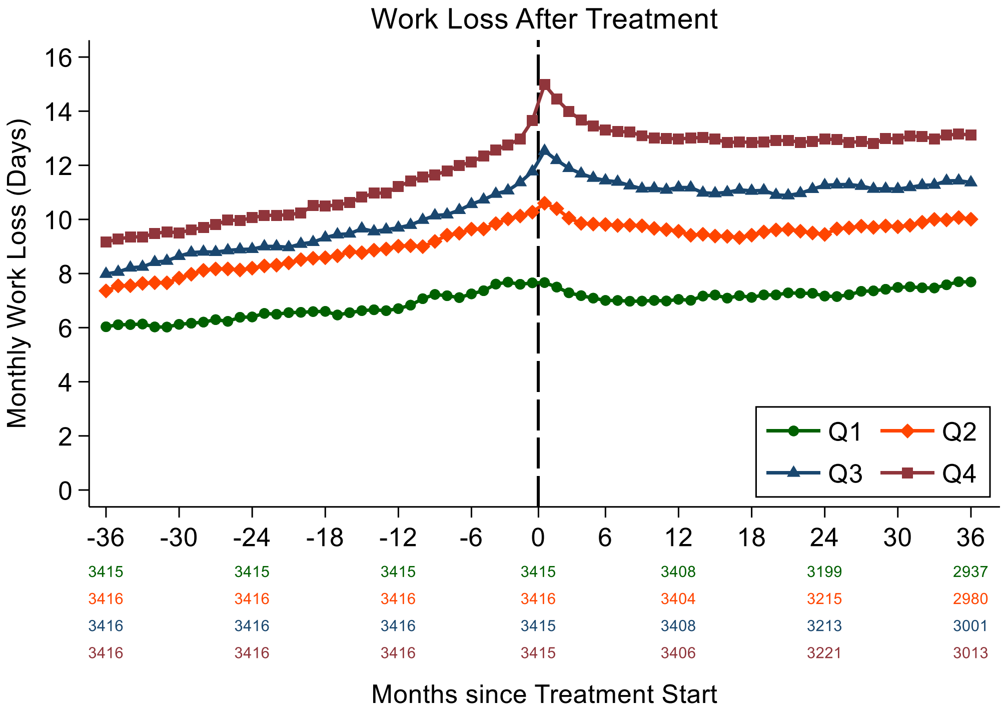

Figure S6 Mean inverse probability weighted monthly work loss in days for patients with RA from three years before to three years after treatment initialisation and VAS pain reported at initiation of drug treatment.

Numbers below x-axis are number of observations for groups of drugs at different points in time.

Observations are weighted by age, sex, education (three levels), year of treatment initiation, and number of previous biologic drug treatments (zero, one, two, three or more).

Patients on the same drug during follow-up (non-switchers)

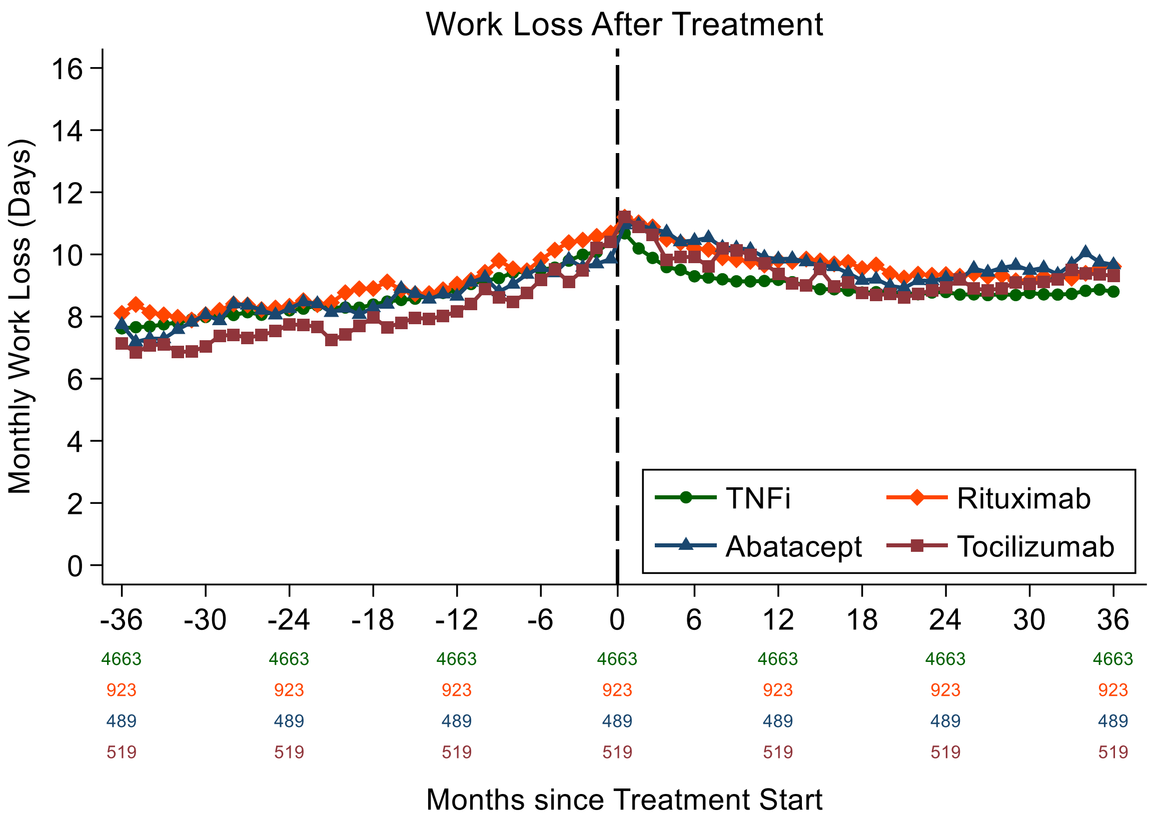

Figure S7 Mean inverse probability weighted monthly work loss in days for patients with RA from three years before to three years after treatment initialisation for patients who remain on the same drug for the whole follow-up period after treatment start.

Numbers below x-axis are number of observations for groups of drugs at different points in time.

Observations are weighted by age, sex, education (three levels), year of treatment initiation, number of work loss days during one, two, or three years prior to treatment initiation, whether or not patient had any work loss days (yes/no) during one, two, or three years prior to treatment initiation, and number of previous biologic drug treatments (zero, one, two, three or more).
